# Prevalence of lymphedema in women undergoing treatment for breast cancer in a referral center in southeastern Brazil

**DOI:** 10.1186/1472-6874-13-6

**Published:** 2013-02-13

**Authors:** Daniella MF Paiva, Vivian O Rodrigues, Marcelle G Cesca, Pamella V Palma, Isabel CG Leite

**Affiliations:** 1 School of Medical Sciences and Health, Juiz de Fora, Brazil; 2 School of Medicine, Institutional Programs for Scientific Start-up Grants (XX PIBIC/UFJF), Federal University of Juiz de Fora, Juiz de Fora, Brazil; 3 School of Dentistry, Institutional Programs for Scientific Start-up Grants (XX PIBIC/UFJF), Federal University of Juiz de Fora, Juiz de Fora, Brazil; 4 Public Health Department of the School of Medicine, Federal University of Juiz de Fora, Juiz de Fora, Brazil

**Keywords:** Lymphedema, Risk factors, Cross-seccional study, Prevalence

## Abstract

**Background:**

Lymphedema is a highly prevalent condition in women who have undergone treatment for breast cancer. Lymphedema negatively affects the quality of life. The objective of this study was to estimate the prevalence of lymphedema and associated factors in women treated for breast cancer in the municipality of Juiz de Fora.

**Methods:**

We performed a cross-sectional study that evaluated 250 women who were being treated for breast cancer. Pre-screening of the sample by analysis of medical records was performed to select women who met the inclusion criteria as follows: women who had an operation more than 6 months ago; absence of active disease, locoregional or distant; the absence of functional change in the affected limb before surgery, which could lead to swelling of the limb; and simulating or masking symptoms of lymphedema, such as bursitis, tendonitis, and work-related musculoskeletal disorders. Women with bilateral breast cancer, absence of axillary intervention (partial or complete axillary dissection and/or SLN biopsy), active disease in the region, or lympho-venous alteration of the limb before surgery were excluded. Data were collected from the medical records of the selected cases, and they subsequently underwent an interview and a physical assessment.

**Results:**

The prevalence of lymphedema was 44.8%. There were medical records on the presence of this condition in 5.4% of cases. With regard to shoulder joint mobility, restrictions on abduction movements, internal and external rotation, and anterior shoulder adduction were significantly associated with lymphedema. Variables, including the presence of seroma, vascular changes, time elapsed after surgery, episodes of redness in the extremities, and cuticle removal from the hand with pliers were considered as major associated factors for lymphedema (p<0.05).

**Conclusions:**

The prevalence of 44.8% for lymphedema found in this study is considered to be relevant because it is a morbidity that produces psychological, physical, and functional damage in patients with this condition. The planning of health programs and services appropriate for the immediate postoperative treatment of women with breast cancer, and increasing the awareness of health professionals regarding the early diagnosis of lymphedema, can help minimize the morbidity of this disease.

## Background

Breast cancer is considered the second leading cause of mortality and morbidity among women in Brazil [1]. The approaches used to control and treat breast cancer have been well studied to the extent that they have led to increased survival of these women [2]. Therefore, there is growing interest in seeking resources and alternatives that could improve the quality of life of breast cancer patients [3-6]. There is also a need to understand the behavior of morbidities from breast cancer that may occur because of tumor development and the therapies used. There is still a high number of late diagnoses that contribute to the election of more aggressive therapy and subsequent traumatic surgery [2,7,8]. This necessitates the improvement of therapeutic techniques to reduce the number of comorbidities presented. Among the notable major innovations are breast surgery techniques and axillary approaches, such as sentinel lymph node (SLN) biopsy [7-10]. Even with the use of these approaches, studies show a high prevalence of postoperative morbidities, mainly regarding lymphedema [7,11-13]. This condition arises from disruption of the lymphatic system in coping with the axillary approach for staging or control of the disease [8,14]. Recent studies on the prevalence of lymphedema and associated factors in specific populations show varying results [8,9,15,16] possibly owing to differing diagnostic methods for detection of lymphedema and to sampling differences. Improving the knowledge regarding risk factors specifically associated with lymphedema in a given population will aid in the formulation of protocols for both its prevention and its treatment, enabling an improved quality of life for these women.

In the municipality of Juiz de Fora, Minas Gerais, southeastern Brazil, there are cancer treatment centers extending services to neighboring micro and macro regions, thus offering a potent sampling source for research in the area.

This study aimed to ascertain the prevalence of lymphedema in women undergoing treatment for breast cancer in a referral unit for cancer treatment in the municipality of Juiz de Fora, as well as associated risk factors. We hypothesized that the prevalence of lymphedema is high and is associated with the time elapsed since surgery and comorbidities among these women.

## Methods

During the period from August 2009 to June 2010, a total of 250 women, who came to the clinic for an oncology (chemotherapy and/or radiotherapy) follow-up visit, were evaluated at a specialized hospital in the municipality of Juiz de Fora. Pre-screening of the sample by analysis of medical records was performed to select women who met the inclusion criteria as follows: women who had an operation more than 6 months ago; absence of active disease, locoregional or distant; the absence of functional change in the affected limb before surgery, which could lead to swelling of the limb; and simulating or masking symptoms of lymphedema, such as bursitis, tendonitis, and work-related musculoskeletal disorders.

All eligible women were invited to participate in the study, and upon signing the consent form, only four women refused to participate. Women with bilateral breast cancer, absence of axillary intervention (partial or complete axillary dissection and/or SLN biopsy), active disease in the region, or lympho-venous alteration of the limb before surgery were excluded.

Data were collected from the medical records of the selected cases, and they subsequently underwent an interview and a physical assessment. Records were analyzed to obtain data regarding the treatment of breast cancer, axillary intervention, seromas, vascular implications, and tumor staging. A semi-structured questionnaire was used for subjective diagnosis of lymphedema and its possible associated factors, followed by the administration of a physical examination for clinical diagnosis and detection of co-existing morbidities. The elapsed time from surgery until the date of the interview was divided into two categories: beyond or within 5 years. Current pain was estimated using the Visual Analog Scale categorized into 10 recording intervals for de-registration.

The physical examination was performed by only one evaluator, who was qualified and trained for this type of approach. The criterion for the diagnosis of lymphedema was obtained by perimetry with the use of a measuring tape, with measurements obtained after adjustment of the dominant arm and time since diagnosis [7,14]. Measurements were taken at seven points: at the inside of the elbow in the supine position (taken as the zero point), and three measurements above and below this point, at intervals of 7.0 cm. Lymphedema was characterized as when the difference between the affected limb and the contralateral (control) limb, using at least one measurement, was equal to or greater than 2.0 cm [7,12,14,17]. This study met the requirements of the Declaration of Helsinki and was approved by the Institutional Ethics Committee of the Federal University of Juiz de Fora (reference number: 050/2009).

### Statistical analysis

The data were processed in a database created with the Statistical Package for Social Sciences version 14.0 (SPSS Inc., Chicago, IL) and Stata 10.0 (StataCorp, Texas). The significance level of the study was 95% (p≤0.05). The presence or absence of lymphedema was considered the dependent variable. The independent variables were aggregated and analyzed in blocks, combined with socio-demographic and health self-assessment variables, characteristics concerning the diagnosis and treatment of cancer, symptoms and presence of Axillary Web Syndrome (AWS) or superficial lymphatic thrombosis in the upper limb ipsilateral to surgery in the postoperative period, information regarding the care and early movement of the limb ipsilateral to surgery, and variables associated with lymphedema in women after surgical treatment of breast cancer. These factors were adjusted for variables on self-reported morbidities, treatment characteristics, and postoperative characteristics. Descriptive analysis was used to obtain absolute and relative frequencies of the analysis variables, as well as for the prevalence of the outcome investigated. Bivariate analysis was performed to assess the association of each independent variable with the dependent variable, using the chi-square (χ^2^) test. In multivariate analysis, Poisson regression was used to analyze the independent variables associated with outcome, controlling for potential confounders, such as the dominant arm and time since diagnosis (adjusted prevalence ratio). The Poisson regression in each block of analysis combined variables with a crude p-value <0.20, and between the blocks, the adopted significance was 5%. The significance level was tested using the Wald test for heterogeneity and linear trend.

## Results

The prevalence of lymphedema in the upper limb ipsilateral to breast surgery was 44.8% (112/250 women). There were medical records on the presence of this condition in 5.4% of cases (6/250 cases). The time elapsed after surgery was significantly associated with the prevalence of lymphedema (p=0.028).

With regard to variables from the postoperative period, the presence of seroma and symptoms, such as redness, were significantly associated with lymphedema (p<0.001) after adjustment by the regression model (Table 1).

**Table 1:** Symptoms and presence of AWS in the arm ispilateral to the surgery, Brazil, 2010

	**Variables**	**%**	***P***
Presence of seroma	No	25.0	<0.001
	Yes	75.0	
Heat Sensation in the scar	No	73.2	0.05
	Yes	26.8	
Pain in the scar	No	48.2	0.013
	Yes	51.8	
Heat sensation in the upper limb	No	63.4	0.015
	Yes	36.6	
Pain in any part of the upper limb	No	27.7	<0.001
	Yes	72.3	
Burning sensation in any part of the upper limb	No	69.6	0.012
	Yes	30.4	
Episode of redness in any part of the upper limb	No	66.1	<0.01
	Yes	33.9	
Presence of lymphatic cord - AWS, anywhere in the upper limb	No	35.7	<0.01
	Yes	63.4	

Variables related to variables for women, such as symptoms and presence of AWS in the arm ispilateral to the surgery in the postoperative period, associated with lymphedema following surgical treatment for breast cancer, Juiz de Fora (MG), Brazil, 2010.

Physical examination showed that 49.6% (124/250) of the women with lymphedema had superficial lymphatic thrombosis, which demonstrated an association with lymphedema in bivariate analysis (p<0.001).

The mean age of patients with lymphedema was 56±11,58 and there was no significant difference in age compared with those without lymphedema (p=0.24). The two groups were similar with respect to variables, such as schooling (p=0.39), obesity (p=0.35), and skin color (p=0.15). In the presence of comorbidities, women with vascular implications (thrombosis, varicose veins, circulatory changes in the lower and/or upper limbs, and others) had a frequency of 2.7 times greater for developing lymphedema (p=0.02). There was no difference between the groups for other self-reported health conditions, including hypertension (p=0.30) and diabetes mellitus (p=0.49, Table 2).

**Table 2:** Women’s socio-demographic and self-reported health characteristics, Brazil, 2010

**Variable**	**Variable category**	**%**	**Crude PR (CI 95%)**	***p***	**Adjusted PR**^**a**^**(CI 95%)**	***p***
Age	up to 56 years	49.1	1	0.17	1	0.20
	57–75 years	50.9	1.30(0.79–2.15)		1.40(0.79–2.45)	
Skin color	non-White	59.8	1	0.05	1	0.15
	White	40.2	1.58(0.94–2.68)		1.51(0.85–2.66)	
Schooling	Secondary/higher	37.5	1	0.13	1	0.39
	Primary	52.7	1.18(0.70–2.00)		1.78(0.55–5.72)	
	Illiterate	9.8	2.24(0.80–6.26)		1.61(0.54–4.79)	
Obesity	No	70.5	1	0.19	1	0.35
	Yes	29.5	1.31(0.77–2.25)		1.31(0.74–2.32)	
Vascular changes	No	95.5	1	0.00	1	0.02
	Yes	4.5	3.84(1.39–10.54)		3.67(1.22–1.11)	

^a^Variables adjusted within their blocks.

Women’s socio-demographic and self-reported health characteristics, associated with the occurence of lymphedema following surgical treatment for breast cancer, Juiz de Fora (MG), \Brazil, 2010.

Women who had undergone surgery more than 5 years previously had a 9.7 times higher frequency of lymphedema compared with those who had undergone surgery less than 5 years previously (p=0.02). The analysis of other variables related to cancer treatment is shown in Table 3.

**Table 3:** Characteristics regarding the diagnosis and treatment of cancer in women, Brazil, 2010

	**Variables**	**%**	**Crude PR(CI 95%)**	***p***	**Adjusted PR**^**a**^**(CI 95%)**	***p***
Staging	0–2	46.4	1	0.001	1	0.216
	3–4	43.8	2.23(1.33–3.72)		1.15(0.78–2.92)	
Type of surgery (breast)	Conservative	40.2	1	0.04	1	0.91
	Radical	59.8	0.63(0.38–1.04)		0.96(0.45–2.00)	
Level of axillary lymphadenectomy	Sampling	6.3	1	<0.01	1	0.52
	Dissection-low	42.0	3.76(1.50–9.42)		1.81(0.53–6.17)	
	Dissection-high	35.7	4.44(1.73–11.40)		1.42(0.39–5.18)	
Chemotheraphy performed	No	11.6	1	<0.01	1	0.69
	Yes	87.5	3.22(1.62–6.38)		4.71(0.62–5.74)	
Any chemotherapy performed on arm ipsilateral to surgery	No	74.1	1	0.11	1	0.91
	Yes	11.6	2.01(0.76–5.29)		1.06(0.34–3.33)	
Radiotherapy performed	No	9.8	1	0.478		
	Yes	90.2	1.12(0.49–2.54)			
Area administered radiotherapy	Biopsy	56.3	1	0.008	1	0.13
	Axilla-breast	28.6	2.67(1.27–5.59)		2.83(0.93–8.56)	
	Breast, axilla, supraclavicular fossa	15.2	2.66(1.17–6.02)		1.52(0.47–4.86)	

^a^Variables adjusted within their blocks.

Characteristics regarding the diagnosis and treatment of cancer in women, associated with the occurence of lymphedema following surgical treatment for breast cancer, Juiz de Fora (MG), Brazil, 2010.

With regard to shoulder joint mobility, restrictions on abduction movements, internal and external rotation, and anterior shoulder adduction were significantly associated with lymphedema (p<0.001).

There was no significant association with outcome for almost all of the variables related to information that the patients received from the healthcare team on caring for their limbs and guidance on early movement after surgery, except for the variable of cuticle removal from the hand with pliers. This variable remained associated with lymphedema even when adjusted for other variables, such as self-reported morbidities and characteristics of the implemented treatment (p=0.02, Table 4).

**Table 4:** Care and early movement of the limb ipsilateral to surgery in women, Brazil, 2010

	**Variables**	**%**	**Crude PR (CI 95%)**	***p***	**Adjusted PR**^**a**^**(CI 95%)**	***p***
Removing hand cuticles with pliers	Yes	40.2	1	0.06	1	0.02
	No	59.8	1.53 (0.92–25)		2.10(1.11–3.95)	
Armpit depilation by razor blade or waxing	Yes	34.8	1	0.10	1	0.81
	No	65.2	1.43 (0.86–2.41)		1.13(0.39–3.31)	
Carry weight with the affected limb	Yes	60.7	1	0.09	1	0.54
	No	39.3	1.47(0.84–20.40)		1.307(0.54–3.11)	
Receive vaccines and/or injections in the limb	Yes	54.5	1	0.08	1	0.96
	No	45.5	1.47(0.88–20.40		1.02(0.39–2.63)	
Injury/burn in any part of the limb	Yes	36.6	1	0.04	1	0.32
	No	63.4	1.58(0.95–2.63)		1.84(0.54–6.28)	
Guidance on exercising in the postoperative period	First day until first month	22.3	1	0.05	1	0.12
	After losing movement	23.2	0.67(0.34–1.31)		1.25(0.61–2.54)	
	Didn’t receive guidance	54.5	(1.67(0.92–3.02)		1.94(0.91–4.18)	

^a^Variables adjusted within their blocks.

Variables related to the clarification received regarding the care and early movement of the limb ipsilateral to surgery in women, associated with lymphedema following surgical treatment for breast cancer Juiz de Fora (MG), Brazil, 2010.

All variables that proved to be significant after adjustment within the block to which they belonged were subjected to Poisson regression to build the final model. Variables that remained significant were vascular disorders (p=0.08), the presence of redness in any region of the arm after surgery (p<0.001), the presence of seroma (p<0.001), and cuticle removal with pliers from the hand corresponding to the limb ipsilateral to surgery (p=0.013), because actual daily routine remained relevant in the model (Table 5).

**Table 5:** Variables associated with lymphedema in women after surgical treatment of breast cancer, Brazil, 2010

	**Variables**	**%**	**Crude PR (CI 95%)**	***p***	**Adjusted PR (CI 95%)**^**a**^	***p***
Vascular pathologies	No	95.5	1	0.02	1	0.08
	Yes	4.5	3.67(1.22–11.11)		3.23(1.06–9.90)	
Seroma	No	25.0	1	<0.01	1	<0.01
	Yes	75.0	3.01(1.61–5.62)		2.71(1.49–4.91)	
Arm redness	No	66.1	1	0.01	1	<0.01
	Yes	33.9	8.11(2.20–29.93)		3.63(1.67–7.87)	
Cuticle removal	No	33.9	1	0.01	1	0.01
	Yes	66.1	2.10(1.116–3.95)		2.07(1.16–3.71)	

^a^ Adjusted between blocks.

Variables associated with lymphedema in women after surgical treatment of breast cancer, after adjustments for variables on self-reported morbidities, treatment characteristics, and postoperative characteristics. Juiz de Fora (MG), Brazil, 2010.

## Discussion

The current study found that the prevalence of patients with lymphedema after treatment for breast cancer was 44.8%. However, it is possible that underestimation occurred because the average time spent in the postoperative period in our sample was 5 years, and the higher a woman’s exposure to risk factors, the greater the chances of developing lymphedema. The possibility of survival bias cannot be ruled out because more severe cases subjected to more drastic measures may not have maintained contact owing to death, and therefore were not included in this sample. The prevalence of lymphedema found in this study is higher than the prevalence rates between 9% and 40% reported by other studies [8,9,12,15,16,18,19]. This difference between prevalence rates could be attributed to the type of method used to diagnose lymphedema, because there are several methods for measuring arm volume, [7,14,17,19] and because of the fact that the institution in which the data were collected does not offer a service specializing in prevention and treatment of lymphedema. Perimetry, which was the diagnostic method used in this study, is based on comparing the measurement of the circumference of the affected arm with the contralateral arm [7,9,14,17]. Furthermore, in Brazil, delayed diagnosis is common, causing more aggressive surgeries, which could be an important reason for the high prevalence of lymphedema in this study.

Age is a variable often associated with lymphedema [7,19-22]. Along with the aging process, anatomical and physiological changes related to lymphatic obstruction occur, which may predispose to the development of lymphedema, with the main mechanism being the opening of lympho-venous anastomoses [7,22-24]. The higher incidence of lymphedema in older patients observed in some studies [7,19,23,25] may be due to a progressive loss of these anastomoses because of the aging process [12,24]. This finding was not observed in the current study, where the average age of the patients with lymphedema was 56 years, with no significant difference between those with and those without lymphedema. Likewise, a study by Yen et al., [20] who investigated self-reporting of the risk factors for lymphedema in older women, also found no significant difference between women with and those without lymphedema. The difference in evaluation methods and the average age of the women involved in these studies may be responsible for the discrepancy of results.

In attempting to reestablish the lympho-venous balance of the upper limb and breast region after breast cancer treatment, the body makes use of compensatory mechanisms, which attempt to avoid edema. However, some factors such as trauma, aging, and repetitive or non-repetitive episodes of infections can overwhelm the lymphatic system, changing the balance. Therefore, the longer the time elapsed since surgery, the greater the risk of developing lymphedema, because this increases the chances of a woman being exposed to injury [17].

Radiotherapy is considered as a risk factor for development of lymphedema, mainly when axillary irradiation is applied [7,13,26]. A likely explanation is the occurrence of lymphedema due to the blockage of lymph vessels or their compression by fibrosis caused by this treatment [25,27,28]. According to Bergmann et al., [7] the main risk factor associated with lymphedema after treatment for breast cancer is the axillary approach, both surgical and radiotherapy, followed by age. Our study differs from previous studies in that there was no association between the irradiation site and the development of morbidity when controlled by other variables.

AWS, otherwise known as superficial lymphatic thrombosis, is the formation of a palpable network of cords that can extend from the armpit, through the antecubital space to the base of the thumb. AWS is caused by possible formation and displacement of fibrin clots in the superficial veins and lymphatic capillaries, which form a network [8,20,29]. In the current study, the presence of AWS in bivariate analysis was statistically significant (p<0.001). However, when AWS was analyzed in the adjusted model, it lost its significance. This syndrome is accompanied by acute pain symptoms that may regress spontaneously (~3 months after surgery) or become chronic, when the formation of a fibrotic cord occurs, and this restricts the range of motion of the shoulder [30,31]. The presence of AWS contributes to the formation or worsening of lymphedema [26,29,31].

Some studies question the validity of current guideline “manuals” for the prevention of lymphedema, because they believe that despite the biological plausibility, no evidence has been shown for the validity of these treatments [6,7,32]. Therefore, it is prudent for the patient to take certain care of the arm, but this should not affect or impair the patient’s daily routine. The occurrence of minor injuries, such as cuts, bruises, minor burns, and infection, triggers an inflammatory response. This translates into an increase of fluid filtered by the arterial capillaries into the interstitium, capillaries and lymphatic vessels, which overloads the lymphatic system already damaged by lymphadenectomy [10,12,20,24]. This process may intensify, generating local symptoms, as well as general symptoms, giving rise to the appearance of erysipelas and/or lymphangitides [20,25,27]. The removal of cuticles during nail care allows easy access to bacteria, by producing a skin lesion, facilitating an inflammatory process that can precede or aggravate lymphedema [4,19,26].

The prevalence of moderate to severe symptoms in women who avoid movement of the ipsilateral arm after surgery is significantly higher than in those who do not avoid it. These data are corroborated by several studies [6,9,16,20] in which changes in movements of the shoulder after surgery for breast cancer were investigated, at various times after surgery, to determine the effect of early exercise intervention through physiotherapy [18,32,33]. In the present study, only 119 women (47.6%) received some type of information concerning various treatments for the limb and the importance of early motion. In this case, besides harm from the lack of intervention supervised by a professional, the guidance of self care alone leads to misinterpretation, resulting in failure to perform adequate exercises. Studies of lymphedema have shown a positive association between the receipt of information and the avoidance of movement [7,16,20,34,35]. In our study, an important association was found between lymphedema and the lack of information regarding movement of the limb, although this was not statistically significant in multivariate analysis.

## Conclusions

Lymphedema is a multifactorial morbidity. Therefore, an interdisciplinary approach is required. The prevalence of 44.8% for lymphedema found in this study is considered relevant because it is a morbidity that produces psychological, physical, and functional damage in patients with this condition. Lymphedema is associated with the time elapsed after surgery, associated vascular pathologies, the presence of seroma, episodes of redness in some parts of the limb after surgery, and cuticle removal with pliers from the hand ipsilateral to the surgery. The surgical method, as well as the extent of the axillary approach, the area administered radiotherapy, and application of chemotherapy show a positive, but not significant, association with the presence of lymphedema.

We conclude that lymphedema is a preventable morbidity. The planning of health programs and services appropriate to the immediate postoperative treatment of women with breast cancer, and increasing the awareness of health professionals regarding the early diagnosis of lymphedema, can help minimize morbidity. Understanding and improved definitions of the associated factors could be important tools for treatment of this condition.

## Abbreviations

AWS: Axillary Web Syndrome; SLN: Sentinel lymph node.

## Competing interests

The authors declare that they have no competing financial interests.

## Authors’ contributions

DMFP conceived the study and participated in the study design, analysis, and drafting of the manuscript. VOR, MGC, and PVP participated in the study design, and performed statistical analysis and data collection. ICG participated in the study design, coordination, and analysis, and drafting of the manuscript. All authors read and approved the final manuscript.

## Authors’ information

DMFP is a professor at the School of Medical Sciences and Health, Juiz de Fora, SUPREMA, Brazil. VOR is a graduate student at the School of Medicine, Institutional Programs for Scientific Start-up Grants (XX PIBIC/UFJF), Federal University of Juiz de Fora, UFJF, Brazil. MGC is a graduate student at the School of Medicine, Institutional Programs for Scientific Start-up Grants (XXII BIC/UFJF), Federal University of Juiz de Fora, UFJF, Brazil. PVP is a graduate student at the School of Dentistry, Institutional Programs for Scientific Start-up Grants (XX PIBIC/UFJF), Federal University of Juiz de Fora, UFJF, Brazil. ICG has a doctorate in public health, and is an adjunct professor of the Public Health Department of the School of Medicine, Federal University of Juiz de Fora, UFJF, Brazil.

## Pre-publication history

The pre-publication history for this paper can be accessed here:

http://www.biomedcentral.com/1472-6874/13/6/prepub
